# Substance use, harm reduction attitudes and behaviors among attendees of nature rave parties in Israel

**DOI:** 10.1186/s12954-023-00845-3

**Published:** 2023-08-09

**Authors:** Hagit Bonny-Noach, Barak Shapira, Pinchas Baumol, Nir Tadmor, Paola Rosca, Stacy Shoshan, Yossi Harel-Fisch, Ariel Caduri

**Affiliations:** 1 Department of Criminology, Ariel University, Ariel, Israel; 2 The Israeli Society of Addiction Medicine, Ramat-Gan, Israel; 3 Ministry of Health, Jerusalem, Israel; 4 Oxygen - Harm Reduction Education, Jerusalem, Israel; 5 Israeli Forum for Safe Spaces, Jerusalem, Israel; 6 Department of Social Work, Tel Hai Academic College, Kiryat Shmona, Israel; 7 Impulse- Integrative Clinical Centre, Haifa, Israel; 8 Department for the Treatment of Substance Abuse, Ministry of Health, Jerusalem, Israel; 9 The Hebrew University of Jerusalem, Jerusalem, Israel; 10 Association for Public Health, Jerusalem, Israel; 11 Sackler Faculty of Medicine, Tel Aviv University, Tel Aviv, Israel; 12 International Research Program on Adolescent Well-Being and Health, Faculty of Education, Bar Ilan University, Ramat Gan, Israel; 13 Israel Authority for Community Safety, Ministry of National Security, Jerusalem, Israel

**Keywords:** Substances use, Harm reduction, Attitudes, Music, Rave, Parties, Nature, Israel

## Abstract

**Background:**

Few studies have analyzed harm reduction behaviors and attitudes among rave party attendees. Since the late 1980s, there has been a large Israeli rave scene, also known as 'Nature Parties'. However, only a few studies have been conducted among nature party attendees and almost all of them are from a qualitative perspective. This study's aim was to fill the gap and conduct quantitative research to investigate the patterns of substance use, harm reduction attitudes and behaviors among Israeli nature rave party attendees.

**Methods:**

A cross-sectional online survey recruited 1,206 people who reported having attended nature rave parties. All of the participants were aged 18–60 years (*M* = 29.9; SD = 7.4), and 770 (64%) were male.

**Results:**

The most common illicit substances used at Israeli nature rave parties in the past year were cannabis (62.2%), followed by LSD (41.4%), MDMA (31.7%), mushrooms/psilocybin (23.9%), ketamine (19.6%) and cocaine (17.2%). A significant but weak association was found between harm reduction behaviors and attitudes toward harm reduction interventions (*r* = .26, *p* < .001) and attitudes toward drug testing kits (*r* = .33, *p* < .001). It seems that although we found higher positive harm reduction attitudes, it is harder to implement harm reduction behaviors. Logistic regressions demonstrated stronger associations with high harm reduction behaviors and higher levels of positive attitudes toward drug testing kits (OR = 4.53; CI 2.97–6.90; *p* < .001), higher levels of positive attitudes toward harm reduction interventions (OR = 4.06; CI 2.62–6.29; *p* < .001), marital status of widower/divorced (OR = 2.22; CI 1.49–3.32; *p* < .001), using MDMA (OR = 1.63; CI 1.19–2.23; *p* <  = .01) and using LSD (OR = 1.41; CI 1.03–1.94; *p* <  = .05).

**Conclusions:**

Formal harm reduction policies and interventions are needed for Israeli nature rave parties in addition to prevention and information programs, which are also very rare. Future studies should examine the subjects of harm reduction attitudes and behaviors among the public, policy makers and professionals.

## Introduction

The rave scene in Israel, also called 'Electronic Dance Music' (EDM), 'Nature', 'forest', 'trance' or 'psytrance' parties, is common and highly attended. The nicknames stem from the location of the parties which is usually outdoors, or after the musical genre that dominates them [1–6].

The Israeli rave scene roots from the late 1980s when it was developed by backpackers returning home from their travels to the state of Goa in India and the islands in Southern Thailand where they were exposed to electronic music parties associated with substance use [1, 7, 8]. In Goa, generations of hippie refugees settled down there starting in the late 1960s. Twenty years later, starting in the late 1980s, electronic parties began in the village of Anjuna creating the unique genre called "Goa Trance". The main attendees at these parties were tourists from Israel, England, Germany, France, and Japan. The drugs used at these parties were primarily LSD and ecstasy (MDMA), sold by the bar owners who also organized the parties [9]. From the parties in Goa and Southern Thailand, the young backpackers brought the partying trend and the music associated with drug use to Europe and Israel [1, 2, 10]. Over the years, and especially from the mid-1990s, a lot of young adult Israelis after completing their mandatory military service travel the 'big trip' to Southeast Asia as part of a rite of passage phenomenon and escapism. They return home and further intensify the Israeli rave scene and substance use [1, 10, 11].

Currently, the most common term for a rave in Israel is 'Nature Party' and there are a variety of different types: 1. Mass mainstream raves involving a very large production. These are most often called festivals where 5000–10,000 partygoers attend and involve licensing and institutional coordination; 2. Medium-sized parties, estimated to be attended by 1000–5000 partygoers; and 3. Small parties attended by hundreds of partygoers, sometimes only dozens for more intimate parties, and are characterized by an underground hue. Most of the underground parties, which are illegal, take place in locations that are hidden, remote, and inaccessible to most, requiring precise instructions to get to the party. Directions to the party are usually only given the day of the party to the participants that registered ahead of time, including voicemail or text messages accompanied by directional signs in the field. The location of the party can be at the beach or in the mountains, desert or forest (sometimes even in prohibited army firing areas), far away from established emergency care facilities. It is estimated that hundreds of small, illegal nature parties take place in Israel every year.

Over the years, the rave scene in Israel has received some research attention mostly from students doing their thesis dissertations in sociology or anthropology. These studies have shown that the rave scene is co-cultural with special values, norms, jargon and rituals. One of the major norms is the use of illicit drugs, mostly MDMA and LSD [1, 4, 12, 13].

Beginning in the 1990s, various studies worldwide reported large amounts of substance use among EDM/rave attendees [14–16]. Raves are considered recreational settings in which drug use is higher than in other settings [17]. For example, among Australian live music event attendees, it was found that the most commonly used drugs in the last 12 months were ecstasy (73.9%), cannabis (64.3%), cocaine (45.5%), hallucinogens (25.3%), amphetamines (21.4%), ketamine (12.9%) and methamphetamines (10%) [16]. Although there is almost no quantitative research among rave attendees in Israel, there is an understanding that raves have contributed to the drug use escalation in Israel [1, 13].

To the best of our knowledge, no previous quantitative studies have characterized Israeli rave scene attendees in the last 20 years. The only other study published examines the use of psychoactive substances in a particular group of 207 parents who are nature rave partygoers. However, this study focused on any time substance use in the preceding year and not only while attending raves [18]. Our study focused on a large number of nature rave attendees and their substance use behavior in their lifetime, the last year, and the last month while attending nature rave parties.

## Harm reduction at raves

Although high substance use among rave attendees is reported in most studies [15, 16], few studies worldwide have analyzed harm reduction behaviors in rave attendees [17]. In Israel, the concept of harm reduction is controversial [19] and, more specifically, the discourse surrounding harm reduction among rave attendees is relatively new. Over the years, Israel has implemented formal harm reduction strategies such as needle and syringe programs for people who inject drugs, and alcohol harm reduction interventions. However, formal harm reduction programs for common types of substances such as cannabis, amphetamine-type stimulants, and hallucinogens are lacking [19]. The classic harm reduction approach that is formally implemented in Israel focuses on the prevention of disease transmission, physical harm, and health promotion.

However, the potential harm from hallucinogens (including LSD and MDMA that act as both stimulants and hallucinogens), which are the most common substances used in nature rave parties, are related mostly to mental health problems, and it is nearly impossible to predict theses harms, if any, to an individual using these substances [12]. Additionally, among party attendees, physical harm may occur due to the substances being taken in high doses and potentially causing toxicity. There is also the potential for dehydration linked with the type of substance used and because of the hot weather during Israeli summers. Over the years, there have been several deaths of young people at nature rave parties published in the media [20, 21]. As a result, informal organizations and volunteers have implemented harm reduction projects and interventions in the form of safety information, consulting and safe zones at nature parties. One of these projects is called "Good People" and was initiated by a youth-in-distress non-governmental organization called ELEM, [12, 19]. "Good People" volunteers identify young people in crisis due to psychoactive substance use at nature rave parties and provide psychological aid and support. In 2018, the "Good People" team reported having treated about 300 emergency cases, with 10% of them in a severe crisis condition [22]. Other volunteer organizations composed mainly of young people inspired by civil society organizations in the USA and Europe implementing harm reduction models have begun to establish safe zones at nature rave parties in Israel as well [19]. In 2016, a group of friends with backgrounds in alternative therapy methods founded "Hof Mivtachìm" (Hebrew for 'safe haven'), and other volunteers established the 'Safe Zone'. These volunteer-based organizations have become more institutionalized, organized, and serious about their work and training during the last few years [12].

In some countries, one approach to harm reduction and health promotion at raves has been providing drug and pill testing kits which allow users to check the content and purity of illicit drugs before deciding to use them. The goal in checking is to potentially reduce drug-related harm [16, 23]. In Israel, the use of such services is prohibited. In this study, we examine the attitudes of nature rave party attendees toward drug testing kits.

As little is known about harm reduction attitudes and behaviors among music rave attendees worldwide, and about the prevalence of substance use among Israeli nature party attendees in particular, the aim of this study was to: (1) identify patterns of substance use among Israeli nature party attendees; (2) explore their attitudes toward harm reduction interventions and toward drug testing kits at nature parties; (3) explore their harm reduction behaviors; and 4) determine whether the socio-demographic background attitude toward harm reduction interventions and drug testing kits and type of substance used is related to harm reduction behaviors.

## Methods

### Participants

Of the 1,260 questionnaires received, a total of 1,206 participants completed the online questionnaire. The inclusion criteria were: a) being at least 18 years old and b) having attended a nature rave party during the last 5 years.

Participants ages ranged from 18 to 60 (*Mean* = 29.9, SD = 7.4), of whom 770 (64%) were male and 432 female (36%). Half of the participants were single (54.1%), almost a quarter in a stable relationship with a partner (23.1%), 17.7% married and 4.7% were divorced or separated. Most of them reported having an academic degree (70.4%) and being secular (69.7%).

### Measures

The questionnaire included:

#### Demographic characteristics and information about participants at nature rave parties

The respondents were asked about their age, gender, family status, religiousness and educational status. Additionally, they were asked about their participation in nature parties such as how many years they have been attending nature parties and at what age they first attended a nature party.

#### Substances used during nature rave parties

The basic structure of the questionnaire was adopted from the 2017 National Epidemiological Survey carried out by the Israel Anti-Drug Authority [24], adjusted by the authors of the present study. The following variables were used to assess reports of substance use during nature parties, all coded as yes or no: (1) cannabis, (2) MDMA, (3) LSD, (4) cocaine, (5) mushrooms/psilocybin, (6) mescaline/cacti, (7) ketamine, (8) laughing gas/ nitrous oxide, (9) DMT, (10) GHB, (11) opioids and (12) alcohol. Participants were asked about their use of these substances during nature parties, in their lifetime, in the last year, and in the last month.

#### Harm reduction attitude questionnaire

This questionnaire was compiled for the purpose of the present study also based upon the questionnaire published by Day et al. [23]. The questionnaire included two parts:

1. Attitudes toward harm reduction interventions during nature parties.
  The questionnaire included 10 items that examined the extent to which it was important for participants that the following interventions would be present during nature parties. For example, a fresh supply of drinking water, a "safe zone" or medical and emergency services. Participants were asked to rate their answers on a Likert 5-point scale, from 1 - "not important at all" to 5 - "essential". Higher mean scores indicated higher levels of positive attitudes toward harm reduction interventions during nature parties. The reliability coefficient was =.82.
2. Attitudes toward drug testing kits.
  The questionnaire included 6 items that examined participants' agreement regarding drug testing kits. For example, 'Drug testing kits will make nature party attendees feel safer' or 'I would like to use drug testing kits at nature parties'. Participants were asked to rate their answers on a Likert scale from 1 - "do not agree at all" to 5 - "largely agree". Higher mean scores indicated higher levels of positive attitudes toward drug testing kits. The reliability coefficient was =.835.

One more question was included about who they think should be responsible for delivering harm reduction interventions and providing information and assistance to attendees using substances. The answer choices were: the nature party attendees, nature party organizers, the police, other authorities, emergency rescue service (in Hebrew, Magen David Adom or MDA) or harm reduction volunteer organizations.

#### Harm reduction behaviors

This part of the questionnaire was compiled for the purpose of the present study and was based on the questionnaire published by Fernández-Calderón, et al., [17]. It included 15-statements examining behaviors of participants during nature parties. For example, 'I mix different drugs' or 'I decide in advance what amount of drugs I intend to consume'. Participants were asked to rate their answers on a Likert scale from 1—" Not at all" to 5—"Always". The Cronbach's Alpha of all items was = 0.78.

### Procedure—study design

We conducted a cross-sectional nationwide online survey. The ethics committee from the first author's IRB approved the research study. After a short pilot study among 30 participants, recruitment advertisements invited nature party attendees to complete an online questionnaire on Israeli rave and nature rave party websites and Facebooks groups. In addition, participants were recruited from WhatsApp groups of nature party attendees who in turn forwarded the questionnaire to other nature party WhatsApp groups, like chain mail. The questionnaire was distributed between May 20 and September 9, 2021 during COVID-19. All participants gave informed consent to participate in this study. Participants were not offered any incentives or asked to provide any identifying personal information. The subjects were assured anonymity and could stop the questionnaire at any time.

### Data analysis

The analyses were carried out using SPSS Version 25. First, the descriptive data were analyzed according to the characteristics of the nature party attendees, substance use, harm reduction attitudes toward harm reduction interventions and toward drug testing kits, and harm reduction behaviors. Cronbach alpha reliability tests were applied to create scales for the harm reduction attitudes and behaviors. The association between harm reduction attitudes toward interventions and drug testing kits and behaviors were analyzed by Pearson's correlation. After transforming the continuous variables to binary variables, logistic regression was employed to identify significant high harm reduction behaviors (the dependent variable) among nature party attendees based on their characteristics, harm reduction attitudes (toward interventions and drug testing kits), and the type of substance used. The categories involving harm reduction attitudes toward potential interventions and the use of drug testing kits were created from ordinal variables numbered 1–5. Hence, higher mean scores indicated higher levels of positive attitudes: "Little" include a mean of 1–2; "Medium" include a mean of 3; and "Lots" include a mean of 4–5.

## Results

The participants were asked about their participation in nature parties. 5.8% reported participating in nature parties during the past year, 43.5% in the past 1–5 years, 27.7% in the past 6–10 years, 15.3% in the past 11–20 years, and 7.7% reported participating for more than 20 years. A quarter (25.5%) reported first attending a nature party before the age of 18, 39.6% between the ages of 18–21, 31.3% between the ages of 22–35, 1.6% between the ages of 36–4,5 and 0.6% over the age of 46 years.

**Fig. 1:**
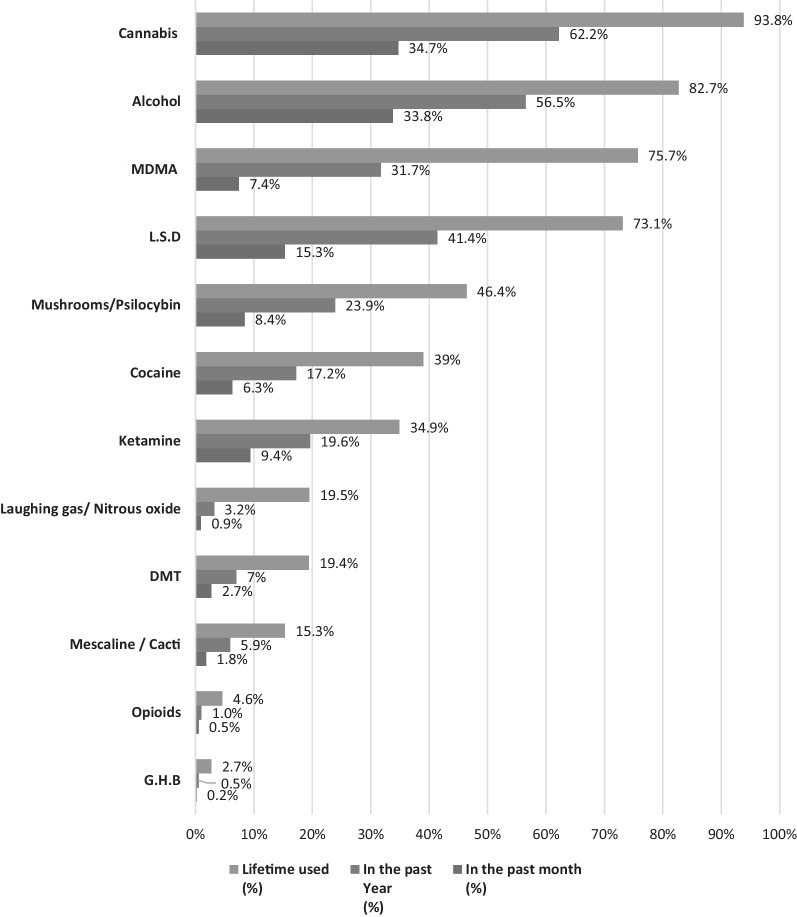
Prevalence of substance use in Israeli nature rave parties among participants: lifetime, past year and past month (*N* = 1,206)

The participants were asked about their substance use during nature parties: lifetime, in the past year and in the past month which is displayed in Fig. 1.

The findings on consumption frequency indicate that the most common illicit substances that had lifetime and past year use at nature parties were cannabis (93.8 and 62.2%, respectively), MDMA (75.7 and 31.7%, respectively), LSD (73.1 and 41.4%, respectively), mushrooms/psilocybin (46.4 and 23.9%, respectively), cocaine (39 and 17.2%, respectively) and ketamine (34.9 and 19.6%, respectively). Additionally, the participants reported high levels of alcohol consumption during nature parties (82.7 and 56.5%, respectively).

Tables 1 and 2 show attitudes toward harm reduction among participants. Table 1 shows the attitudes toward harm reduction interventions during nature parties.

**Table 1:** Attitudes toward harm reduction interventions during nature parties among participants (*N* = 1,206)

To what extent is it important to you,if at all, that the following things are available at nature parties?	Not at all/slightly important (%)	Important (%)	Important to a large extent/essential (%)
Fresh supply of drinking water	1.8	4.1	94.1
Cooling: water spray and shading	2.4	6.4	91.2
Medical and emergency services (such as medical and ambulance personnel)	2.6	7.5	89.8
"Safe zone"—a quiet area to rest that provides support as needed	4.4	7.5	88.2
Mental health professionals (such as social workers)	12.4	14.7	73
Access to information about the effects and harms of using different substances	16	16.4	67.6
Drug testing kits	26	12.3	61.7
Contraceptives (such as condoms)	37.4	20.4	42.2
Earplugs	35.2	27.1	37.7
Security cameras	56.5	19.2	24.3

**Table 2:** Attitudes toward drug testing kits among nature party attendees (*N* = 1,206)

To what extent do you agree with the following statements regarding drug testing kits?	Not at all/minimally agree (%)	I do not have a clear opinion (%)	Agree/largely agree (%)
Drug testing kits will make nature party attendees feel more safe	7.2	10.9	81.9
I think drug testing kits are important for harm reduction at nature parties	6.7	11.4	81.9
I think drug testing kits will be useful	7.8	11.6	80.5
I would like to use drug testing kits at nature parties	10.4	15.1	74.4
Drug testing kits should be provided free of charge at nature parties	12	20.1	68
Drug testing kits will cause party attendees to consume more substances*	69	23.1	7.9

*-Reverse question

The results in Table 1 reveal that a large proportion of participants support harm reduction interventions and show high positive attitudes toward harm reduction interventions (Mean = 3.72, SD = 0.69).

Table 2 presents the attitudes of the participants toward drug testing kits.

The results in Table 2 reveal that a large proportion of participants support the use of drug testing kits and show high positive attitudes toward drug testing kits (Mean = 4.06, SD = 0.77).

The participants were asked their opinion about who they think should be responsible for delivering harm reduction interventions, providing information and assisting the nature party attendees while using substances. The results showed that most of the participants (73.5%) think that party attendees should be responsible, followed by party organizers (67.8%), other state authorities (49.2%), harm reduction organizations (46.9%), and lastly the police and MDA (27.2%).

Table 3 shows harm reduction behaviors among the nature party attendees.

**Table 3:** Harm reduction behaviors among nature rave party attendees (*N* = 1,206)

To what extent does each of the following statements happen to you at nature parties?	Not at all/a little (%)	Sometimes(%)	A lot/always (%)
Before substance use, I research and read about the substance I plan to consume at the party	24	15.3	60.7
I do not mix different drugs during nature parties*	14.1	30.3	55.5
I do not mix alcohol with other drugs during nature parties*	24.6	25.2	50.1
When I mix substances, I use the information table about the safety of mixing substances or other information sources	62.3	12.7	25
I decide in advance what amounts of substance I intend to consume at the party	28.7	24.2	47.1
If a friend gives me a substance at a nature party, I do not trust him*	37.9	31.6	30.6
During the party I take proactive breaks from the dance floor to rest	15.1	21.3	63.6
I make sure to drink enough water during the nature party	3.4	10	86.7
I use additives like electrolytes or magnesium, during nature parties	65.8	16.3	17.9
I use substances at a party when I know what it is and what it’s source is	17.5	19.9	62.6
On the day / two days after the party I make sure to rest	19.7	25.8	54.5
I only use substances in an atmosphere that is comfortable for me at the party (comfortable setting)	10.6	12.3	77.2
I only use substances at a party if I feel good (comfortable setting)	9.2	12.7	78.1
I make sure to have a good friend with me at the party who accompanies me and keeps me from any trouble that may come	13.4	17.2	69.4
I plan in advance what substances I will consume at the party	19.1	21.9	58.9

*-Reverse question

The results in Table 3 reveal that a lot of the participants reported high engagement in harm reduction behaviors (Mean = 3.46, SD = 0.61).

Pearson's correlation examined the association between harm reduction attitudes and behaviors among participants. There was a significant but weak positive correlation between attitudes toward harm reduction interventions during raves and harm reduction behaviors (*r* = 0.26, *p* < 0.001). In other words, the higher the positive attitude toward harm reduction interventions during raves, the higher the harm reduction behavior level. Also, there was a significant but weakly positive correlation between attitudes toward drug testing kits and harm reduction behaviors (*r* = 0.33, *p* < 0.001), so the higher positive attitudes toward drug testing kits, the higher the harm reduction behavior level.

The logistic regression results, used to identify high vs. low engagement in harm reduction behaviors among participants at raves, are presented in Table 4.

**Table 4:** Logistic regression to identify high engagement in harm reduction behaviors in nature party attendees (*N* = 1206)

Variable	Value	OR	95% C.I. for	
			Lower	Upper
*Socio-demographics*				
Gender	Women-1, Men-0	1.13	0.84	1.50
Age group	26–31-1, 18–25-0	0.85	0.58	1.24
	32–60-1, 18–25-0	0.73	0.47	1.14
Marital status	Married-1, Single-0	1.84	0.97	3.48
	Divorced/separated-1, Single-0	2.22***	1.49	3.32
Education	Diploma-1, High school-0	1.24	0.79	1.96
	BA student-1, High school-0	0.89	0.59	1.34
	BA-1, High school-0	1.21	0.79	1.87
	MA-1, High school-0	1.23	0.70	2.16
Nagelkerke *R*^2^ = 0.035				
*Attitudes toward harm reduction*				
Positive attitudes	Little-1, No-0	2.15***	1.35	3.43
	Medium-1, No-0	3.36***	2.18	5.17
	Lots-1, No-0	4.06***	2.62	6.29
Nagelkerke *R*^2^ = 0.062				
*Attitudes toward drug testing kits*				
Positive attitudes	Little-1, No-0	1.94**	1.21	3.11
	Medium-1, No-0	2.52***	1.62	3.92
	Lots-1, No-0	4.53***	2.97	6.90
Nagelkerke *R*^2^ = 0.074				
*Substances use at nature parties in the past year*				
Cannabis	Yes-1, No-0	1.34	0.97	1.84
Alcohol	Yes-1, No-0	0.60***	0.45	0.81
MDMA	Yes-1, No-0	1.63**	1.19	2.23
L.S.D	Yes-1, No-0	1.41*	1.03	1.94
Mushrooms/psilocybin	Yes-1, No-0	0.77	0.54	1.09
Cocaine	Yes-1, No-0	0.58*	0.38	0.89
Ketamine	Yes-1, No-0	0.94	0.63	1.39
Laughing gas/nitrous oxide	Yes-1, No-0	0.27*	0.08	0.90
Nagelkerke *R*^2^ = 0.055				

*p < .05, **p < .01,***p < .001

The socio-demographic characteristics added to the univariate models include gender, marital status, education, and three age groups of relatively equal size. It should be noted that the choice of the comparison group categories is based upon previous requirements by the sponsoring ministry—centering on people aged 18–25 that represent the core of Israel's military and reserve service. Moreover, dividing each continuous or ordinal variable into categories allowed us to obtain odd ratios. These ratios reflect each category's importance to policymakers.

The odd ratios (OR) show that harm reduction behaviors had stronger associations with positive attitudes toward drug testing kits (OR = 4.53; CI 2.97–6.90; *p* < 0.001), with positive attitudes toward harm reduction interventions (OR = 4.06; CI 2.62–6.29; *p* < 0.001), with certain demographic characteristics and behaviors, such as being a divorced/separated (OR = 2.22; CI 1.49–3.32; *p* < 0.001), using MDMA (OR = 1.63; CI 1.19–2.23; *p* <  = 0.01) and using LSD (OR = 1.41; CI 1.03–1.94; *p* <  = 0.05). Additionally, the OR was low for alcohol use, and very significant, suggesting negative attitudes toward harm reduction (OR = 0.60; CI 0.45–0.81; *p* <  = 0.001).

## Discussion

This study aimed to investigate the patterns of substance use, harm reduction attitudes and behaviors among Israeli nature party attendees.

In accordance with previous studies reporting that rave settings are characterized by higher rates of substance use compared to other settings [15–17], also our findings reveal that Israeli nature party attendees reported much higher rates of substance use compared to the general Israeli population, according to the latest national epidemiological survey [24]. Although methodologies were different in the two studies, the findings of the present study reveal a very large difference between substance use among nature party attendees compared to that of the general population. For example, rates of cannabis use over the past year in the national survey were 27% compared to 62.2% in our study, MDMA 0.58% compared to 31.7%, LSD 0.77% compared to 41.4%, mushrooms/ psilocybin 0.3% compared to 23.9%, and cocaine 0.5% compared to 17.2%, respectively.

Our findings reveal that the most common illicit substances used at nature parties in the past year were cannabis (62.2%), followed by LSD (41.4%), MDMA (31.7%), mushrooms/ psilocybin (23.9%), ketamine (19.6%) and cocaine (17.2%). Our study's results differ from those of studies in other countries. For example, other studies found that ecstasy/MDMA is the most commonly used substance among EDM attendees in Australia [16] and New York [14]. It also seems that hallucinogenic substances, mostly LSD & mushrooms/psilocybin, are dominant at nature parties in Israel, which is also in contrast to other studies [16].

This trend of using LSD, ketamine, psilocybin and MDMA coincides with the global surge of research and therapy known as the Psychedelic Renaissance [25]. In Israel, there has been a change is perception and usage of these substances as local researchers are studying the effects of MDMA, psilocybin and other psychedelics. There have also been shifts in Israel regarding access to cannabis and ketamine as they have become legal for "medical use." Moreover, public opinion regarding the safety and use of these substances has changed over time.

As few studies worldwide have analyzed harm reduction behaviors among rave attendees [17], our study expands the knowledge concerning harm reduction behaviors and attitudes and examines for the first time Israeli nature party attendees' characteristics and attitudes. Although in Israel harm reduction strategies are controversial [19], and the discourse of harm reduction among nature party attendees is relatively new, the findings of our study reveal that a large proportion of participants support harm reduction interventions. A lot of the participants in this study were also familiar with the volunteer organizations that provide safety information, consultation and safe zones at nature parties [12, 19]. In Israel, the use of drug and pill testing services for substance users are prohibited and nature party attendees consider the current police activities at nature parties as a barrier to adopting harm reduction services, in particular drug testing kits [26]. However, the participants were familiar with these services and support their use even more than other harm reduction interventions.

The findings in this study reveal a significant but weak correlation between harm reduction attitudes toward drug testing kits and interventions and harm reduction behaviors. It seems that although there are higher positive harm reduction attitudes, it is harder to implement harm reduction behaviors. More than half of the participants reported that they do not mix different drugs (55.5%) and half reported that they do not mix alcohol with other drugs (50%). Nevertheless, logistic regression demonstrated that those with more positive attitudes toward harm reduction drug testing kits and with more positive attitudes toward harm reduction interventions in general, had higher odds of engaging in harm reduction behaviors. In light of this, it is recommended to increase their positive attitudes toward harm reduction to further increase their harm reduction behaviors.

The most highly supported harm reduction behavior among participants was 'to drink enough water' (87%). It seems that nature party attendees consider drinking water to be one of the most important measures to reduce physical harm that can occur due to raised body temperature and dehydration caused by certain substances, such as MDMA [27], in combination with the hot weather during the Israeli summer. Interestingly, our results showed that those who used MDMA reported the highest harm reduction behaviors compared to those who used other substances.

Additionally, the logistic regression demonstrated a stronger association with high harm reduction behaviors with marital status, particularly among divorced/separated individuals compared to singles. Since Israeli culture places such a strong emphasis on children [28] and most of these individuals have children, it is possible that the dependence of their children on only one parent could indicate a greater sense of responsibility toward their own well-being. However, there is currently a lack of studies to support this idea. Therefore, further research is necessary, particularly on a larger and more diverse sample of divorced individuals, in order to understand their motivation to engage in harm reduction behaviors.

The findings reveal that nature party attendees who reported using MDMA and LSD are associated with more harm reduction behaviors compared to those who use other substances. This finding is not surprising, since LSD and MDMA are perceived to potentially cause mental harm [12]. In addition, MDMA is also perceived as a substance that can potentially cause physical harm such as dehydration [27]. Many people who consume ecstasy are aware that they might consume a substance which is not purely or exclusively MDMA [29], which is why most drug testing kits are designed to test for the existence of adulterants.

Additionally, a significant association with harm reduction behaviors was found among those who use alcohol during nature parties, although the OR was not high. It seems that attendees are aware of the fact that mixing alcohol with other substances is not recommended because it can be dangerous [17]. Much work has been done by harm reduction volunteers, advocates and organizations in disseminating safety information and consulting as well as at safe zone at nature parties [12, 19].

Most of the participants are highly cognizant of the need to minimize harm from their drug use. Furthermore, they believe that other nature party attendees and the party organizers should be responsible for delivering harm reduction interventions and providing information and assistance to nature parties attendees who are using substances. Information and interventions for harm reduction strategies should be accessible and distinctly intended for partygoers so that they better know how to manage situations as needed. Also, it should be clear to party organizers that it is their responsibilities in assisting and ensuring implementation of harm reduction interventions during nature parties.

Moreover, participants believe that they need to take care of themselves in the context of harm reduction interventions and not solely trust the authorities, and especially not to rely on the police[26] and emergency medical services possibly because they consider those services as less approachable.

The participants opinions about who they think should be responsible for delivering harm reduction interventions reflect the lack of organized policies and services surrounding harm reduction strategies. Harm reduction strategies should be formally adopted in Israel because harm reduction is still controversial [19] and much depends on trust and policing [26]. As testing kits are also illegal in Israel, there is no other way than to formally change policymakers' opinions and change regulations and laws to accommodate harm reduction interventions in Israel.

### Limitation

Due to the self-reported nature of the cross-sectional online survey, our findings may not be representative of all of the nature party attendees in Israel. Additionally, our questionnaire included only a limited amount of background information about patterns of participation in nature parties.

## Conclusions

Formal harm reduction policies and interventions are needed in Israel to better address the needs of nature party attendees, in addition to the implementation of large-scale prevention and information programs. Future research, quantitative as well as qualitative, is needed to understand and investigate harm reduction behaviors, beliefs, and perceptions among nature party attendees, as well as among the public, policy makers and professionals.

## Data Availability

The database for this study is not publicly available. It could be available from the corresponding author on reasonable request.

## Abbreviations

COVID-19: Coronavirus disease of 2019
DMT: Dimethyltryptamine
EDM: Electronic dance music
GHB: Gamma hydroxybutyrate
LSD: Lysergic acid diethylamide
MDA: Emergency rescue service
MDMA: 3,4-Methylenedioxy methamphetamine
OMT: Opioid maintenance treatment
OR: Odds ratio
